# P-923. Aminoglycosides as a Carbapenem-Sparing Strategy for Urinary Tract Infection Caused by Extended-Spectrum Beta-Lactamase Producing Enterobacterales: A Retrospective Comparative Study

**DOI:** 10.1093/ofid/ofaf695.1129

**Published:** 2026-01-11

**Authors:** Curtis Sera, Niki Arab, Brian Kim, Chelsea Morinishi, Bryant Yang, Arthur Jeng

**Affiliations:** Olive View-UCLA Medical Center, Sylmar, California; Olive View- UCLA Medical Center, Los Angeles, California; Olive View-UCLA Medical Center, Sylmar, California; David Geffen School of Medicine at UCLA, Los Angeles, California; UCLA, Los Angeles, California; Olive View UCLA Medical Center/UCLA School of Medicine, Sylmar, California

## Abstract

**Background:**

The rise of extended spectrum beta-lactamase (ESBL) producing Enterobacterales has led to increased carbapenem (CP), raising concerns for carbapenem resistant Enterobacterales (CRE) and *Pseudomonas* (CRP), in addition to increasing *Clostridium difficile* infection (CDI) risk due to gut microbiome disruption. Aminoglycosides (AG) lack these risks and remain a viable treatment (tx) for ESBL producing Enterobacterales. At Olive View-UCLA Medical Center (Sylmar, CA), AGs were deployed as a CP-sparing strategy for ESBL-related urinary tract infection (UTI). The aim of this study was to evaluate the use of AG vs CB for ESBL Enterobacterales UTIs.

**Figure 1:**
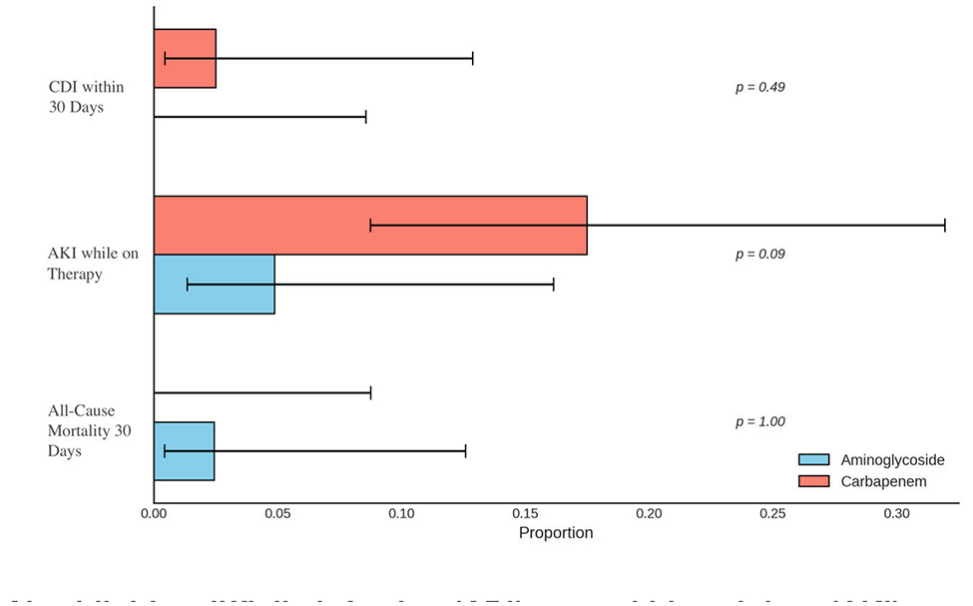
Secondary outcomes Clotridioides difficile infection (CDI), acute kidney injury (AKI)

**Methods:**

This single center retrospective study from 1/2019-5/2023 included adults ≥18 years of age admitted for UTI. All positive urine cultures (Ucx) with ESBL *E. coli, Klebsiella,* or *Proteus* spp were reviewed and included if treated with CP or AG for >3 days (d) for either cystitis or pyelonephritis. Primary outcomes were tx success (no recurrent UTI with the same organism within 30d) and symptom resolution by discharge. Secondary outcomes were 30d all-cause mortality, tx discontinuation due to side effect (SE), acute kidney injury (AKI), CDI incidence within 30d, and CRE or CRP infection within 90d. Primary outcomes were compared using one sided z-test for non-inferiority (5% margin) and secondary outcomes with Fisher’s exact test using R (R Core team, 2024).

**Results:**

Of 540 Ucx reviewed, 81 met criteria (41 AG, 40 CP). The CP group had higher baseline creatinine and more frequent bacteremia. Otherwise, there was no difference in baseline characteristics [Table 1]. AG was non-inferior to CP for treatment success (90.2% vs. 77.5%; difference 12.7%, 95% CI: -0.53% to 26.0%; p = 0.014) and improvement by discharge (97.6% vs. 90.0%; difference 7.6%, 95% CI: -1.19% to 16.3%; p = 0.009). There were no differences in secondary outcomes [Figure1]. One AG case developed CRP. None in either group developed CRE within 90d of therapy or stopped tx due to SE.

**Conclusion:**

AG were non-inferior and had more favorable outcomes compared to CP for ESBL UTI treatment. Both showed similar safety profiles, with no increased risk of AKI or other side effects. Findings support AG as a viable CP-sparing alternative in ESBL UTIs.

**Disclosures:**

All Authors: No reported disclosures

